# Overexpression of HER2 in the pancreas promotes development of intraductal papillary mucinous neoplasms in mice

**DOI:** 10.1038/s41598-018-24375-2

**Published:** 2018-04-18

**Authors:** Wataru Shibata, Hiroto Kinoshita, Yohko Hikiba, Takeshi Sato, Yasuaki Ishii, Soichiro Sue, Makoto Sugimori, Nobumi Suzuki, Kosuke Sakitani, Hideaki Ijichi, Ryutaro Mori, Itaru Endo, Shin Maeda

**Affiliations:** 1 Department of Gastroenterology, Department of Medicine, Yokohama City University Graduate School of Medicine, Yokohama, Japan; 2 Advanced Medical Research Center, Yokohama City University, Yokohama, Japan; 3 Department of Gastroenterology, Department of Medicine, University of Tokyo, Tokyo, Japan; 4 Institute for Adult Diseases, Asahi Life Foundation, Tokyo, Japan; 5 Department of Gastroenterological Surgery, Yokohama City University Graduate School of Medicine, Yokohama, Japan

## Abstract

Pancreatic ductal adenocarcinoma (PDA) has a 5-year survival rate of less than 5% and is the sixth leading cause of cancer death. Although *KRAS* mutations are one of the major driver mutations in PDA, *KRAS* mutation alone is not sufficient to induce invasive pancreatic cancer in mice model. *HER2*, also known as *ERBB2*, is a receptor tyrosine kinase, and overexpression of *HER2* is associated with poor clinical outcomes in pancreatic cancer. However, no report has shown whether *HER2* and its downstream signaling contributes to the pancreatic cancer development. By immunohistochemical analysis in human cases, HER2 protein expression was detected in 40% of PDAs and 29% of intraductal papillary mucinous carcinomas, another type of pancreatic cancer. In a mouse model, we showed overexpression of activated *HER2* (*HER2*^*NT*^) in the pancreas, in which cystic neoplastic lesions resembling intraductal papillary mucinous neoplasm-like lesions in humans had developed. We also found that *HER2*^*NT*^ cooperated with oncogenic *Kras* to accelerate the development of pancreatic intraepithelial neoplasms. In addition, using pancreatic organoids in 3D cultures, we found that organoids cultured from *HER2*^*NT*^*/Kras* double transgenic mice showed proliferative potential and tumorigenic ability cooperatively. HER2-signaling inhibition was suggested to be an new therapeutic target in some types of PDAs.

## Introduction

Pancreatic ductal adenocarcinoma (PDA) has a 5-year survival rate of less than 5% and is the sixth leading cause of cancer death^1^. Although *KRAS* mutations are one of the major driver mutations in PDA, *KRAS* mutation alone is not sufficient to induce invasive pancreatic cancer in mice model^2–5^.

Human epidermal growth factor-2 (*HER2*; also known as *ERBB2*) is a 185 kDa receptor tyrosine kinase, and a point mutation in its transmembrane domain causes malignant transformation^6^. It was reported that overexpression of activated *HER2* under control of the MMTV promoter led to mammary adenocarcinoma in a single step, suggesting that downstream signaling activated by *HER2* drives carcinogenesis in certain tissues^7^. *HER2*-targeted therapy is now an standard treatment for breast and gastric cancers with *HER2* amplification^8–10^, and overexpression of *HER2* has been associated with poor prognosis in pancreatic cancer. However, no study has been shown how *HER2* alone or with *Kras* mutation is involved in the development of pancreatic neoplasms in genetically engineered mouse models.

In this study, we showed that pancreas-specific overexpression of activated *HER2* in mice led to intraductal papillary mucinous neoplasm (IPMN)-like lesions. We also assessed a role of activated *HER2* in *Kras*-driven pancreatic neoplasms, by using mice harboring activated *HER2* and/or *Kras* mutation in pancreas^11^, and found that a cooperative role between activated *HER2* and oncogenic *Kras* accelerated the development of pancreatic intraepithelial neoplasms.

## Results

### HER2 protein expression in surgically resected human PDA and IPMC

**Figure 1:**
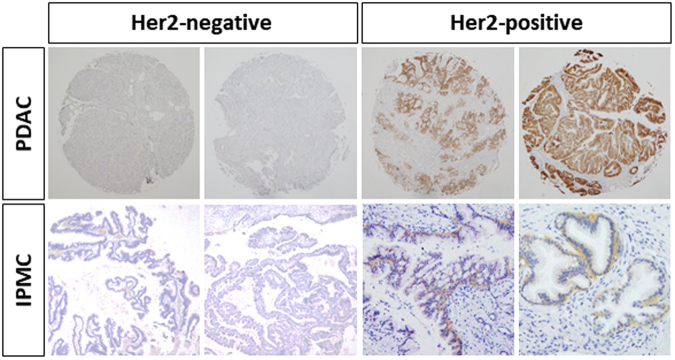
Representative image of *HER2* immunohistochemistry in surgically resected human PDA or IPMC. Immunohistochemical staining of *HER2* in PDA (tissue array) or surgically resected IPMC (original magnification 100×).

To evaluate HER2 protein expression in human PDA, we used a human tissue array consisting of human PDA tissues from 20 patients and 8 normal pancreatic tissues. By immunohistochemical analysis, we detected strong HER2 expression in 8 (40%) human PDA cases, whereas no HER2 expression was found in the normal tissues (Fig. 1). We also collected surgically resected human IPMC specimens and assessed the expression of HER2 protein by immunohistochemistry (Fig. 1). Among 31 resected IPMC specimens, 9 (29%) were strongly positive for HER2 protein expression. Clinical parameters (patient age and sex; tumor location, subtype, grade, and size; and serum tumor markers) showed no significant differences between HER2-positive and -negative IPMCs (Table 1).

**Table 1:** Clinical characteristics according to HER2 status.

		HER2(+) (n = 9)	HER2(−) (n = 22)	*p* value
Age (median)	70 (50–80) years	68	70	n.s.
Sex (M/F)		5/4	20/2	n.s.
Stage	Non-invasive	3	8	n.s.
	Minimally invasive	2	5	
	Invasive	4	9	
Location	Head	4	10	n.s.
	Body	3	10	
	Tail	0	1	
	Head, body and tail	2	1	
Duct type	Main duct	6	8	n.s.
	Branch duct	2	3	
	Mixed	1	11	
Size (median)		37	40	n.s.
Tumor marker (median)	CEA (ng/ml)	4.2	2.1	n.s.
	CA19–9 (U/ml)	18	15	n.s.

### HER2-induced IPMN-like lesions in mouse pancreas

**Figure 2:**
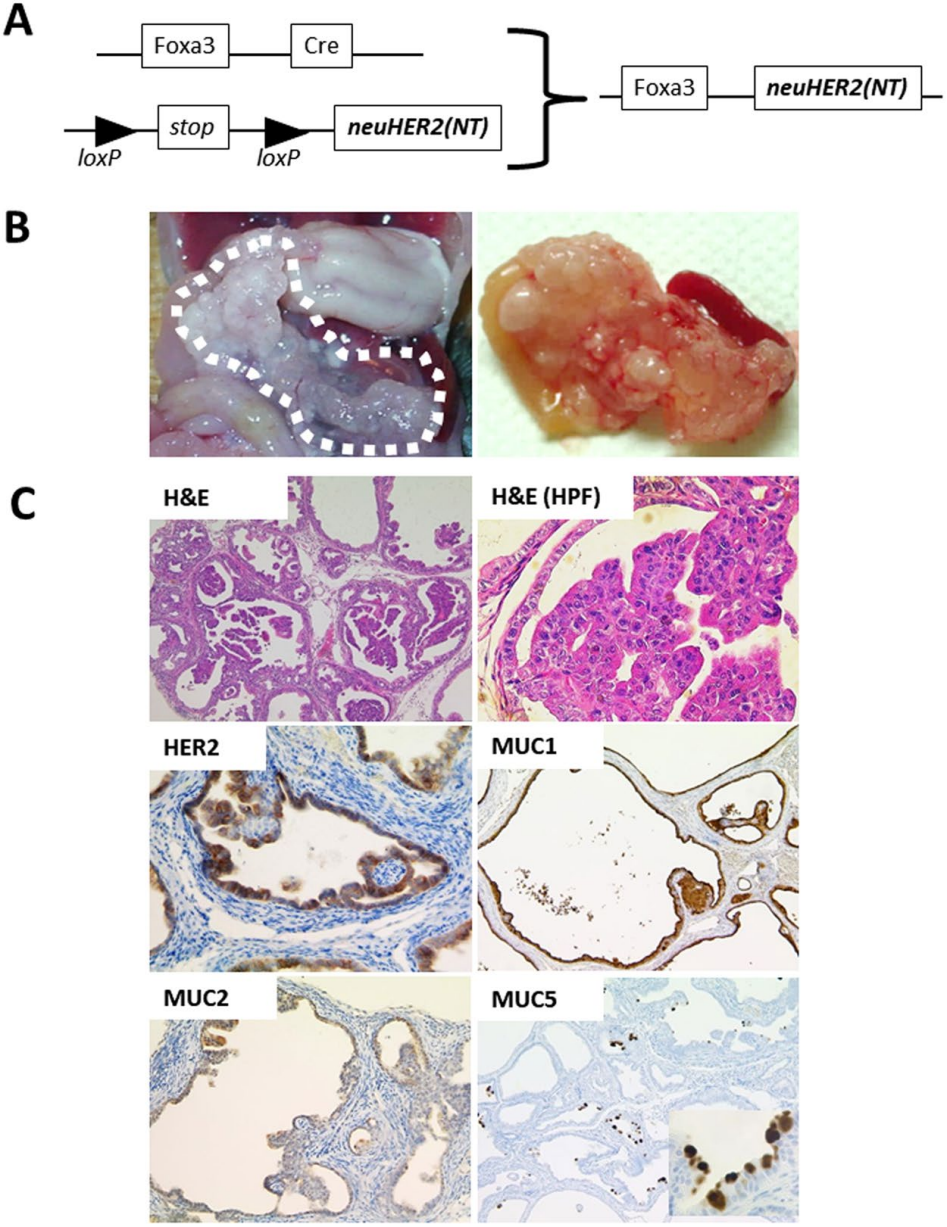
Conditional *HER2*^*NT*^ expression in mouse tissue. (**A**) Construction of HER2NT transgenic mice. Foxa3-Cre mice were crossed with lox-stop-lox-HER2NT mice. (**B**) Representative gross image and pathology in 12-week-old Foxa3-Cre;HER2NT mice. (**C**) Representative images of immunohistochemical staining of HER2, MUC1, MUC2, and MUC5 and the corresponding H&E staining. Original magnification 100×, and 400× (HPF).

**Figure 3:**
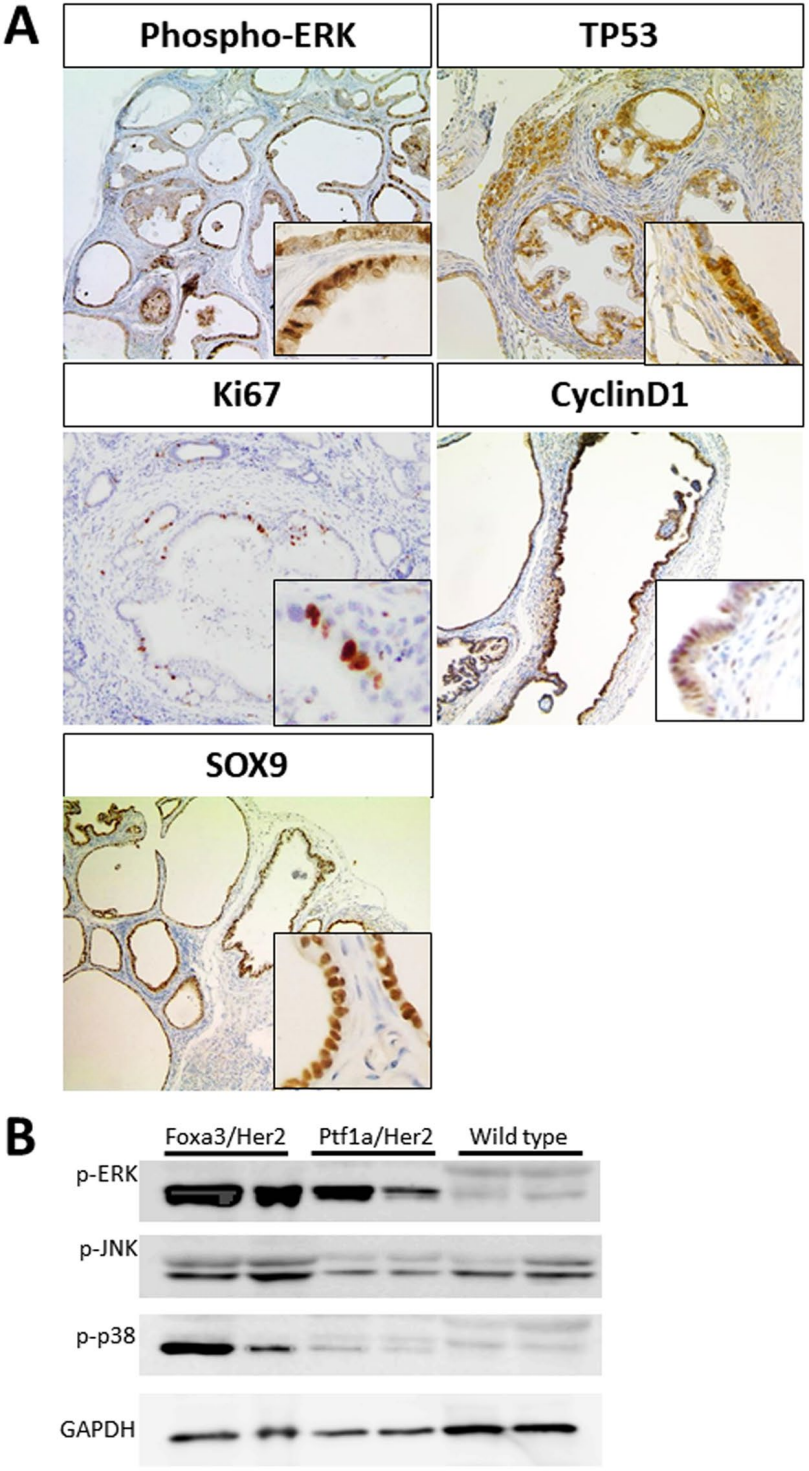
Characterization of cystic lesions in *HER2*^*NT*^ mouse tissue. (**A**) Immunohistochemical staining in cystic lesions in *Foxa3*-*Cre*;*HER2*^*NT*^ mice. The antibodies used for immunostaining are indicated. Original magnification 100×. (**B**) Immunoblot analysis of MAPK signaling pathway molecules in *Foxa3-Cre*;*HER2*^*NT*^, *Ptf1a-Cre*;*HER2*^*NT*^, and wild type mouse pancreas.

As shown above, we found that 30–40% of pancreatic tumors were positive for HER2. To investigate the pathogenic role of HER2 in pancreatic biology, we established a mouse model by crossing *LSL*-*HER2*^*NT*^ with *Foxa3-Cre* mice (*Foxa3-Cre*;*HER2*^*NT*^) (Fig. 2A). Foxa3 is reportedly expressed in endoderm vertical pancreatic bud during the early embryonic stage^12^. To clarify which cells express Cre recombinase, we crossed *Foxa3-Cre* with *Rosa26-YFP* mice; in resulting mice, we detected YFP expression in almost all pancreatic acinar cells but not in beta cells (Figure S1). At 8 weeks of age, *Foxa3-Cre*;*HER2*^*NT*^ mice developed cystic lesions exhibiting papillary proliferation in almost all pancreatic tissue (Fig. 2B). H&E staining showed loss of acinar cells in the pancreatic parenchyma, broad cystic changes, and elevated papillary lesions. Papillary epithelial cells showed dysplasia of low- to high-grades, resembling human IPMN with focal high-grade dysplasia (Fig. 2C). Immunohistochemical analysis confirmed HER2 expression in epithelial cells of the cystic lumen (Fig. 2C). To characterize the cystic lesions, we performed immunohistochemistry and found strong expression of MUC1 and MUC5 and weak expression of MUC2, which are markers of the pancreatobiliary and oncocytic IPMN types in humans (Fig. 2C). The activation of ERK, a critical downstream molecule of HER2, was strong in cystic epithelial cells (Figs 3A and S3). We also detected strong nuclear TP53 staining in the epithelial cells of cystic lesions, suggesting that these lesions have malignant potential (Fig. 3A). To assess the cell proliferation status in cystic lesions, we performed immunohistochemical staining of Ki67 and cyclin D1. While the wild type control showed weak staining for both markers, we detected many Ki67- and cyclin D1-positive cells in cystic lesions, suggesting that these cystic lesions develop a high proliferative potential following overexpression of *HER2*^*NT*^ (Fig. 3A). SOX9, a marker of pancreatic ductal stem cells^13–15^, was strongly expressed in cells of the cystic wall, suggesting a pancreatic ductal lineage of the cystic lesions. To assess the intracellular signaling pathways downstream of *HER2*^*NT*^, we performed immunoblot analysis in pancreatic tissues. Compared with wild-type mice, both *Foxa3-Cre*;*HER2*^*NT*^ and *Ptf1a-Cre;HER2*^*NT*^ mice showed the activation of downstream MAPK signaling, including ERK, JNK, and p38, as determined by the phosphorylation level of each protein (Fig. 3B). All of these results suggest that HER2 signaling contributes to the development of IPMN via activation of canonical MAPK signaling in pancreas.

### HER2 expression accelerates murine pancreatic intraepithelial neoplasia formation in KRAS-mutant mice

**Figure 4:**
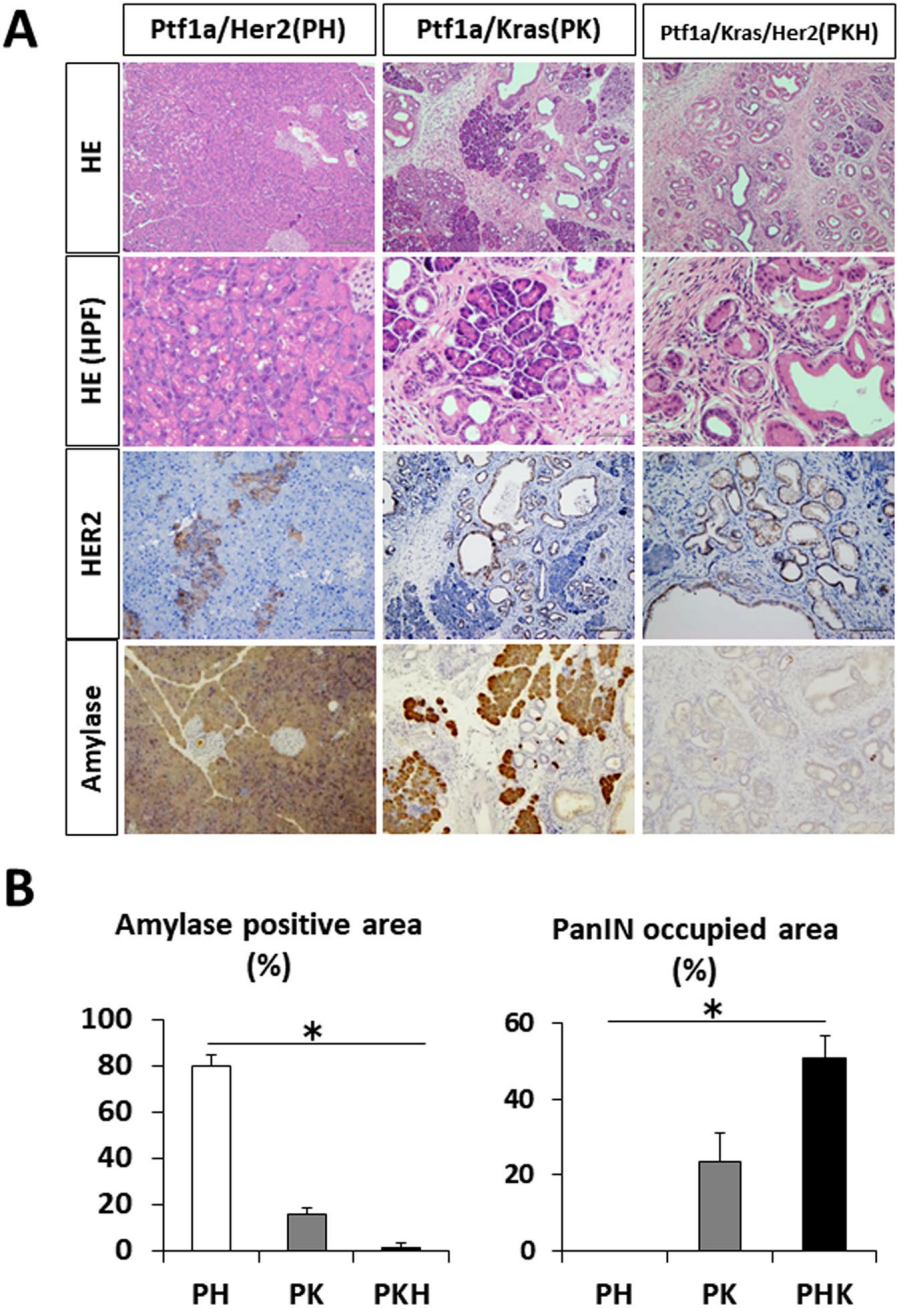
Histological sections from 24-week-old *Ptf1a-Cre*;*HER2*^*NT*^, *Ptf1a-Cre*;*Kras*, or *Ptf1a-Cre*;*Kras*;*HER2*^*NT*^ mice. (**A**) H&E staining and immunohistochemical staining of HER2 and amylase. Original magnification 100×, and 200× (HPF). (**B**) Percentages of amylase-positive or PanIN areas in each mouse.

Since a large number of acinar cells were lost in *Foxa3-Cre*;*HER2*^*NT*^ mice, most mice have died within 10 weeks after birth. Because 10 weeks was not enough time to analyze the role of *HER2*^*NT*^ in pancreatic carcinogenesis, we used *Ptf1a-Cre* mice to assess the role of *HER2*^*NT*^ in pancreas. Surprisingly, *Ptf1a*;*HER2*^*NT*^ (PH) mice showed almost no phenotypes in pancreas at 6 months of age. Immunohistochemical analysis showed HER2 expression in 20–30% of pancreatic acinar cells (Fig. 4A). The activation of ERK, JNK, and p38 was weak in PH mouse pancreas compared with *Foxa3-Cre*;*HER2*^*NT*^ mouse pancreas (Figs 3B and S3). These results suggest that weak HER2 expression in PH mouse pancreas may be one reason for the difference in phenotype compared with *Foxa3-Cre*;*HER2*^*NT*^ mice, in which *HER2* was under the control of a different Cre-promoter. In accordance with this, MUC1, MUC2, and MUC5 expression was negative in PH pancreas. In addition, all acinar cells were amylase positive, and the number of Ki67-positive cells was similar to that in the control mice (Figure S2).

Since over 90% of human PDAs exhibit *KRAS* mutation^16^, we assessed the role of *HER2*^*NT*^ in *Kras*-mutant mice. We crossed *HER2*^*NT*^ mice with *Ptf1a-Cre*;*Kras* mice, which express mutant *Kras* or *HER2*^*NT*^ or both *Kras* and *HER2*^*NT*^ in the pancreas, and the resulting mice were sacrificed at 6 months old to evaluate the phenotype of the pancreas. At 6 months old, we found severe acinar cell loss in *Ptf1a-Cre*;*Kras*;*HER2*^*NT*^ (PKH) mice and mild acinar cell loss in *Ptf1a-Cre*;*Kras* (PK) mice (Fig. 4A). In PK mice, murine pancreatic intraepithelial neoplasia (mPanIN)-1 lesions were distributed widely throughout the pancreas, whereas mPanIN-2 or −3 lesions were rarely found, as described previously^5^. Interestingly, PKH mice showed accelerated mPanIN lesions with severe acinar cell loss compared with PH mice (Fig. 4A,B). The majority of these lesions were still predominantly mPanIN-1. However, a few more mPanIN-2 and mPanIN-3 lesions were also observed. The number of TP53-positive cells was increased in the pancreas of PKH mice compared with PK mice, whereas the numbers of Ki67 and SOX9-positive cells did not differ (Figure S2). These results suggest that overexpression of HER2 not only plays a role in IPMN development, but is also important in PanIN-PDA carcinogenesis.

### Tumorigenic properties of organoid-derived tumors

**Figure 5:**
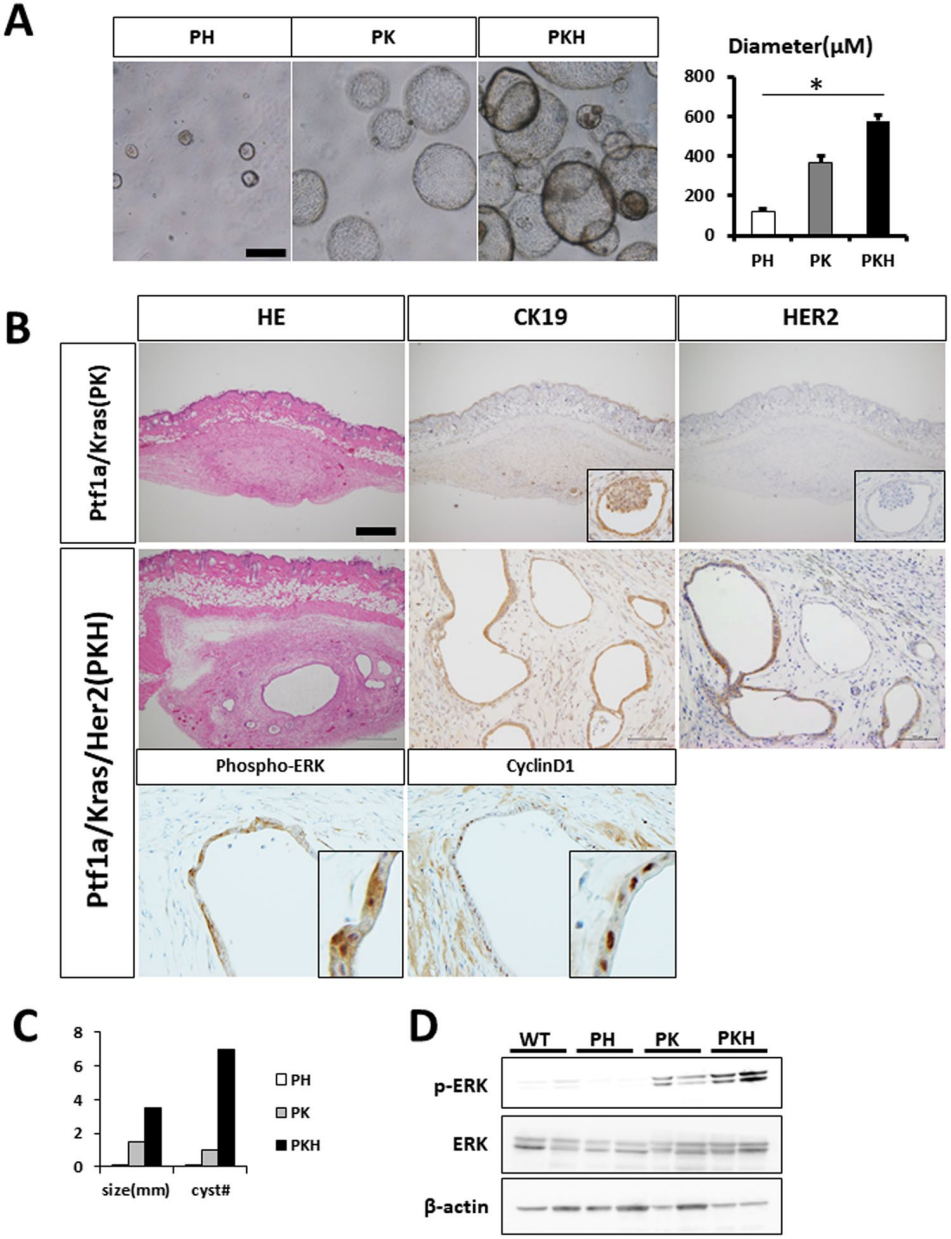
Synergy between *Kras* and *HER2*^*NT*^ activation in organoids for tumorigenesis. (**A**) Representative images and diameters of organoid cultures from each mouse: *Ptfa-Cre1*;*HER2*^*NT*^ (PH), *Ptf1a-Cre*;*Kras* (PK), or *Ptf1a-Cre*;*Kras*;*HER2*^*NT*^ (PKH). (**B**) Subcutaneous tumor sections from nude mice. H & E staining and immunohistochemical staining of CK19 and HER2 in PK and PKH tumors (upper panel). Immunohistochemical staining of phosphorylated ERK and cyclin D1 in PKH tumors (lower panel). (**C**) Size and number of cysts in subcutaneous tumor sections (n = 3 per strain). (**D**) Immunoblot analysis of phosphorylated ERK, ERK, and β-actin in organoids.

Since the murine pancreatic organotypic culture system is reportedly useful for analyzing the molecular properties of pancreatic cancer development^11,17^, we cultured organoids originating from wild-type and transgenic mice (PH, PK, or PKH mice; Fig. 5A). After 7 days, the size and numbers of PH organoids were similar to those of wild-type organoids (data not shown). After 4 weeks of culture, wild type and PH organoids were collected and injected into nude mice. At 3 weeks after inoculation, we did not detect any tumors in either group. These results suggested that *Ptf1a*-Cre driven *HER2*^*NT*^ organoids did not exhibit tumorigenicity in nude mice (data not shown). Next we cultured PK and PKH organoids and found that they were increased in size and number compared with wild-type and PH organoids (Fig. 5A). Moreover, PKH organoids were increased in size compared with PK organoids (Fig. 5A). PK and PKH organoids were collected and injected into nude mice. At 3 weeks post-inoculation, we detected subcutaneous lesions in nude mice injected with organoids from both the PK and PKH mice (Fig. 5B). We performed immunohistochemical staining of these tumors and confirmed that the tumors were positive for HER2 and the epithelial marker CK19 (Fig. 5B). Compared with tumors from PK mice, those from PKH mice were larger with many cystic structures (Fig. 5C), suggesting that *HER2*^*NT*^ cooperates with *Kras* to exert tumorigenic properties. In accordance with these results showing activation of downstream signaling pathways, ERK was strongly activated, as confirmed by its phosphorylation, in PKH organoids compared with PK organoids (Figs 5D and S4). In order to test the growth inhibition of organoids by HER2 inhibitor, we used Lapatinib, and measured OD450 value at before and after 72 hours of administration. After adding Lapatinib, both Her2- and Her2/Kras- organoid showed the growth inhibition after adding Lapatinib (Figure S5).

### Effectiveness of HER2 inhibition in human pancreatic cell lines

**Figure 6:**
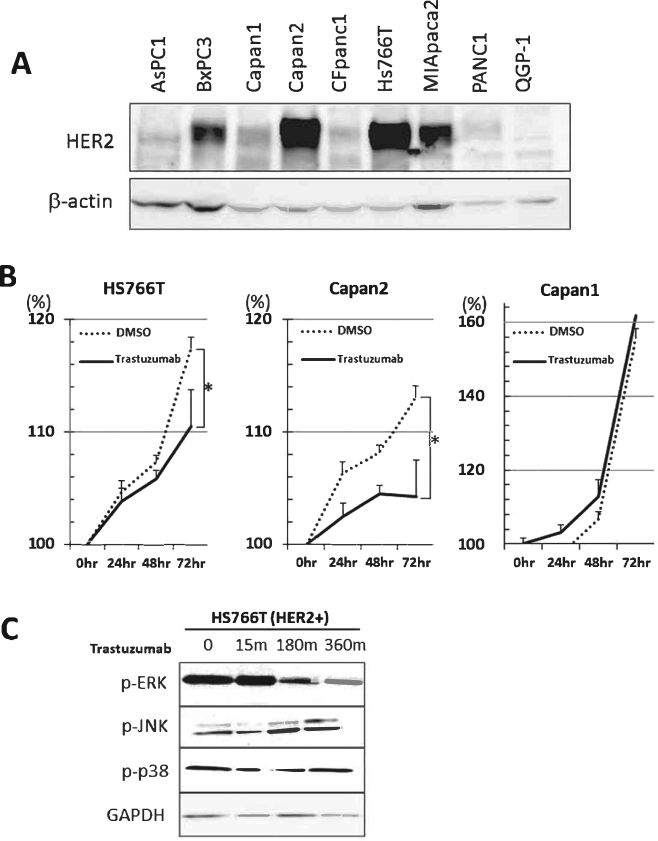
*In vitro* analysis of HER2 inhibition in human pancreatic cancer cells. (**A**) Immunoblot analysis of HER2 in nine pancreatic cancer cell lines. β-actin was used as an internal control. **(B**) Cell proliferation in pancreatic cancer cells with or without trastuzumab treatment. Trastuzumab (10 µg/100 cells) was added, and living cells were measured using a WST8 cell proliferation assay kit. (**C**) Immunoblot analysis of MAPK signaling in HS766T pancreatic cancer cells. The time points and antibodies are indicated. GAPDH was used as an internal control.

Next, we analyzed HER2 expression in several human pancreatic cell lines. Among nine cell lines, four were strongly positive for HER2 expression (Figs 6A and S6). We evaluated the effect of the HER-neutralizing antibody trastuzumab on the proliferation of HER2-positive Capan2 and Hs766T cells and HER2-negative Capan1 cells. Among those cell lines, HER2-positive Capan2 and Hs766T cells were sensitive to HER2 inhibition, whereas HER2 inhibition was not effective in HER2-negative Capan1 (Fig. 6B). We also confirmed that ERK, but not JNK or p38, activation was inhibited by HER2 inhibition (Figs 6C and S7). These results suggest that HER2 expression determines the effectiveness of a HER2-neutralizing antibody.

## Discussion

While *KRAS* mutations are the most well-known and evidence-based driver mutations in PDA, no driver mutation or predictive marker has been identified in IPMC, especially for determining prognosis or whether to perform surgical resection. We demonstrated here that forced expression of *HER2*^*NT*^ in the pancreas gave rise to IPMC-like lesions in the pancreas, and *HER2*^*NT*^ accelerated acinar cell loss in *Kras* mice, which might accelerate the conversion of pancreatic ductal cells into PDA.

Genomic mutations in human PDA and IPMC have been well demonstrated^2,3,18^. Among 13 different *KRAS* mutations in PDA, 3 (G12D, G12R, and G12V) comprise the majority of all *KRAS* mutations detected in PDA. The other mutations occurred in *SMAD4*, *TP53* and *p16*, and no other mutations have been detected in our work^16^. As for human solid cancers, amplification, rather than mutation, of *HER2* has mainly been detected in human gastric cancer tissue^19^. In PDA, only a few cases show *HER2* mutation or amplification^2^, whereas more than 50% of PDA cases are positive for HER2 protein expression^20^, suggesting a discrepancy between genetic alteration and protein expression of HER2.

In accordance with human genomic data, many mouse models of PDA have been developed using mutant *Kras* since 2003^4,5,21,22^. Several reports have also described the development of IPMN-like lesions in mouse models^23–26^. In the current study, we used mutant *HER2*^*NT*^ to force expression of *HER2*^*NT*^ in the pancreas of mice and detected IPMN-like lesions. Similar to our *HER2*^*NT*^ mice, Bardeesy *et al*. reported that the combination of *Kras*^G12D^ with *Smad4* deletion resulted in the rapid development of IPMN, although selective deletion of *Smad4* alone in pancreatic epithelial cells showed no distinct phenotype^23^. TGF-alpha, which is an upstream component of the EGFR signaling pathway, also contributed to the IPMN phenotype in mice in combination with *Kras*G12D. TGF-alpha seemed to activate STAT3 signaling, resulting in inhibition of apoptosis and contributing to development of the IPMN phenotype^25^. Figura *et al*. reported that loss of *Brg1* by mutation of *Kras* resulted in human IPMN-like lesions, implicating a distinct mechanism in IPMN development other than the Kras-driven carcinogenic pathway^27^.

Different *Cre*-expressing mice are useful to determine cellular origins in mouse models. In the current study, we used *Foxa3-Cre* or *Ptf1a-Cre* mice, and the former was reported to express in endodermal cells of the hindgut at E8.5^28^. Cre expression was capable of causing IPMN by forced expression of *HER2*^*NT*^ alone, whereas *Ptf1a-Cre*, which is expressed at E9.5^29^, did not cause IPMN lesion development, suggesting that the cell type or duration of *Cre* expression is strongly associated with the development of IPMN. Further analysis such as lineage tracing should be performed using tamoxifen-driven *Cre*-expressing mice to determine the cellular origin.

After the introduction of trastuzumab, the use of molecular targeted therapies with detection of molecular markers such as HER2 has increased in the field of cancer therapy. In real-world practice, trastuzumab is an effective drug for patients with gastric cancer^10^; when *HER2* positivity is confirmed, and no exclusion criteria are detected, patients are treated with trastuzumab. Although other molecular targeted therapies are rapidly being approved for solid tumors including pancreatic cancer, clinical trials of trastuzumab for pancreatic cancer were not successful^30^. However, since it is an effective drug for other cancers, a method of selecting the appropriate patients for trastuzumab treatment is needed. Based on our current finding that 30–40% of PDA/IPMN patients were positive for HER2, trastuzumab therapy can be used for patients expressing HER2-dependent signaling pathway molecules, not just HER2 itself, determined by cancer tissue, and if possible, by culturing primary cancer cells from patients^11^.

In the current study, we established 3D organotypic cultures to evaluate tumorigenicity in nude mice using a xenograft model. Since 3D cultures result in more precise and accurate biological phenomena compared with 2D cancer cell cultures, it was beneficial to screen for effective cancer treatment drugs preclinically^11^. In the near future, it would be worth banking the primary cell line with organoids and using them for drug screening before testing the drug directly on patients.

In conclusion, we generated a novel murine IPMN model by crossing *HER2*^*NT*^ mice with conventional *Kras*^G12D^ mice. Overexpression of *HER2*^*NT*^ may induce not only IPMN in the pancreas but also rapid acinar cell loss and PanIN formation in *Kras*^G12D^-mice, suggesting that acceleration of *HER2*^*NT*^*-*driven signaling could contribute to the development of human PDA/IPMN. Inhibition of *HER2* in pancreatic neoplasms may be a therapeutic option for certain types of PDA/IPMN.

## Methods

### Mice

All protocols for animal experiments were approved by the Committee for Animal Experiments at the Yokohama City University, Yokohama, Japan (approval number #F-A-14–043). All methods were carried out in accordance with relevant guidelines and regulations.

We generated conditional transgenic mice expressing a mutant form of activated rat HER2 (*HER2*^*NT*^) using the lox-stop-lox system (*HER2*^*NT*^ mice). Constitutive expression of *HER2*^*NT*^ was achieved following deletion of the stop element by expression of *Cre*-recombinase. The *HER2*^*NT*^ sequence was excised from the plasmid pSV2-*HER2*^*NT*^, which was kindly gifted by RA Weinberg, and then digested by HindIII and SalI and subcloned into the pEGFP-C2 vector (Clontech Laboratory Inc., Mountain View, CA, USA). Using KpnI and SacI, this construct was inserted into pCALNL5 (RIKEN BRC, Tsukuba, Japan) to create the LSL-*HER2*^*NT*^ vector. The final construct was sequenced before injection into mice (data not shown). Potential founder mice were screened by PCR. Two mouse strains exhibiting high levels of *HER2*^*NT*^ expression were selected and backcrossed with C57BL/6 J mice. There were no histological alterations in other organs, including the intestine, liver, lung, and kidney, of LSL-*HER2*^*NT*^ mice (data not shown). LSL-*HER2*^*NT*^ mice were crossed with *Cre*-harboring mice.

Transgenic founder mice harboring LSL-*HER2*^*NT*^ were mated with *Foxa3-Cre* mice^31^ or *Ptf1a-Cre* mice^29^ to generate pancreas-specific *HER2*^*NT*^ expressing mice. To analyze a role of activated *HER2*^*NT*^ in *Kras*-driven pancreatic carcinogenesis, we crossed *Ptf1a-Cre* mice with LSL-*HER2*^*NT*^ mice and/or *LSL-Kras*^*G12D/*+^ mice^32^.

### Human pancreatic cancer tissue

This study protocol employing tissue specimens of human IPMC was approved by the Internal Review Board at Yokohama City University, Yokohama, Japan (Approval number #B150108031). Informed consent had been obtained by enrolled patients with giving a chance to opt out from the study at any time. All methods were performed in accordance with the relevant guidelines and regulations. We used surplus formalin-fixed paraffin embedded (FFPE) tissue samples of human intraductal papillary mucinous carcinoma (IPMC) which were surgically resected and diagnosed at the Department of Surgery, Yokohama City University, Yokohama, Japan. The human PDA tissue array was purchased commercially from BioMax (Rockville, MD, USA).

### Immunohistochemical analysis

Histopathological analysis was performed by using hematoxylin and eosin (H&E) staining. Immunohistochemical analysis of *HER2* was performed to evaluate cell proliferation and downstream intracellular signaling in neoplastic tissue. Primary antibodies used in this study were anti-HER2 (1:200, CST#4290, Cell Signaling Technology, Danvers, MA, USA), anti-MUC1 (1:100, ab15481, Abcam, Cambridge, MA, USA), anti-MUC2 (1:200, sc-15334, Santa Cruz Biotechnology, Santa Cruz, CA, USA), anti-MUC5 (1:100, sc-21701, Santa Cruz Biotechnology), anti-Ki67 (1:100, ab16667, Abcam), anti-CyclinD1 (1:25, CST#2978, Cell Signaling Technology), anti-phospho-p44/p42 (1:100, CST#4376, Cell Signaling Technology), anti-SOX-9 (1:100, sc-20095, Santa Cruz Biotechnology), anti-TP53 (1:200, CST#2524, Cell Signaling Technology), and anti-CK19 (1:50, sc-376126, Santa Cruz Biotechnology).

### IPMN subtypes

Using immunohistochemical analysis, the IPMN subtypes were determined as follows. The gastric subtype consisted of cells expressing MUC5AC but not MUC1 or MUC2. The intestinal type consisted of cells expressing MUC2 and MUC5AC but not MUC1. The pancreatobiliary type consisted of cells expressing MUC1 and MUC5AC but not MUC2. The oncocytic-type consisted of cells with abundant eosinophilic cytoplasm and focal expression of MUC5AC and MUC1 but no expression of MUC2.

### Immunoblot analysis

We evaluated the activation of downstream intracellular signaling, such as ERK, JNK, p38, and AKT, in mouse pancreatic tissue using immunoblot analysis. The primary antibodies used for immunoblotting were anti-GAPDH (1:1000, CST#2118, Cell Signaling Technology), anti-phospho-ERK (1:1000, CST#4376), anti-phospho-JNK (1:1000, CST#4668), and anti-phospho-p38 (1:1000, CST#4511).

### Three-dimensional (3D) organotypic culture of pancreatic cells

We cultured pancreatic cells in Matrigel (Corning #354248, Corning, NY, USA) in serum-free medium for establishment of 3D organoids as described previously^11,33,34^. Briefly, mouse pancreatic tissue was minced and dissociated by collagenase P (10 mg/ml; Roche, Basel, Switzerland) using dispase for 30 min at 37 °C. We used 100-μm cell strainers to remove tissue debris. The tissue material was centrifuged and resuspended, and isolated pancreatic cells were embedded in 30 μl Matrigel followed by culturing in medium (AdDMEM/F12) supplemented with HEPES (Invitrogen, Carlsbad, CA, USA), Glutamax (Invitrogen), 1 mM N-acetylcysteine (Sigma-Aldrich, St. Louis, Missouri), 100 ng/ml Wnt3A (R&D Systems, Minneapolis, MN, USA), 1 mg/ml R-spondin1, 100 ng/ml noggin (Peprotech, Rocky Hill, NJ, USA), 50 ng/ml epidermal growth factor (Peprotech), 10 nM gastrin (Sigma), 100 ng/ml fibroblast growth factor 10 (Peprotech), 10 mM nicotinamide (Sigma), and 0.5 μM A83–01 (Tocris Bioscience, Bristol, UK).

### Xenograft model of organoid-derived tumor cells

Organoids were maintained in Matrigel for 4 weeks and then injected subcutaneously into 6-week-old male mice (KSN/Slc nude mice, Japan SLC, Shizuoka, Japan). Four weeks after injection, the tumors were subjected to the experiments.

### Reagents

We used two HER2 inhibitors; anti-human HER2 monoclonal antibody (Chugai Pharma, Japan) and Lapatinib (GW-572016, Selleck, USA) for the experiments *in vitro*. Cell Counting Kit-8 (WST-8, Dojindo, Japan) was used to assess cell proliferation as manufactures instruction.

### Statistical analysis

Comparisons of the clinical characteristics according to HER2 status were performed using Fisher’s exact test (Excel Statistics, Social Survey Research Information Co., Ltd, Japan). Other experimental data were analyzed by Student’s t-test or Kruskal–Wallis analysis.

## Electronic supplementary material

Supplementary Information
