# Supplementary material for: Overexpression of HER2 in the pancreas promotes development of intraductal papillary mucinous neoplasms in mice
Source: Sci Rep. 2018 Apr 18;8:6150. doi: 10.1038/s41598-018-24375-2 (PMC5906617; doi:10.1038/s41598-018-24375-2)

**Supplementary Information**

Overexpression of HER2 in the pancreas promotes development of intraductal papillary mucinous neoplasms in mice

Wataru Shibata^1,2^, Hiroto Kinoshita^3^, Yohko Hikiba^4^, Takeshi Sato^1^, Yasuaki Ishii^1^, Soichiro Sue^1^, Makoto Sugimori^1^, Nobumi Suzuki^4^, Kosuke Sakitani^4^, Hideaki Ijichi^3^, Ryutaro Mori^5^, Itaru Endo^5^, and Shin Maeda^1^

1. Department of Gastroenterology, Department of Medicine, Yokohama City University Graduate School of Medicine, Yokohama, Japan
2. Advanced Medical Research Center, Yokohama City University, Yokohama, Japan
3. Department of Gastroenterology, Department of Medicine, University of Tokyo, Tokyo, Japan
4. Institute for Adult Diseases, Asahi Life Foundation, Tokyo, Japan
5. Department of Gastroenterological Surgery, Yokohama City University Graduate School of Medicine, Yokohama, Japan

**Figure S1. Confirmation of Cre expression in the pancreas and stomach.** Immunohistochemical staining of YFP in *Foxa3cre*;LSL-YFP mice. Original magnification 100×.

**Figure S2. Immunohistochemical analysis in 24-week-old *Ptf1a-Cre*;*HER2^NT^*, *Ptf1a-Cre*;*Kras*, or *Ptf1a-Cre*;*Kras*;*HER2^NT^* mice.** Immunohistochemical staining of MUC1, MUC2, MUC5, Ki67, TP53, and SOX9. Original magnification 100×.

**Figure S3. Characterization of cystic lesions in *HER2^NT^* mouse tissue.**

A. Immunohistochemical staining in cystic lesions in *Foxa3*-*Cre*;*HER2^NT^* mice. The antibodies used for immunostaining are indicated. Original magnification 100×. B. Immunoblot analysis of MAPK signaling pathway molecules in *Foxa3*;*HER2^NT^*, *Ptf1a*;*HER2^NT^*, and wild type mouse pancreas.

**Figure S4. Synergy between *Kras* and *HER2^NT^* activation in organoids for tumorigenesis**. Immunoblot analysis of phosphorylated ERK, ERK, and β-actin in organoids.

**Figure S5. (top)** Representative micrographs of organoids. Organoids from Her2/Kras mice was treated with either Lapatinib by the concentration indicated (100x, original magnification).

**(bottom)** Absolute value of WST-8 assay 72 hours after administration with Lapatinib.

**Figure S6. Immunoblot analysis of HER2 in nine pancreatic cancer cell lines.** β-actin was used as an internal control.

**Figure S7. *In vitro* analysis of HER2 inhibition in human pancreatic cancer cells.** Immunoblot analysis of MAPK signaling in HS766T pancreatic cancer cells. The time points and antibodies are indicated. GAPDH was used as an internal control.


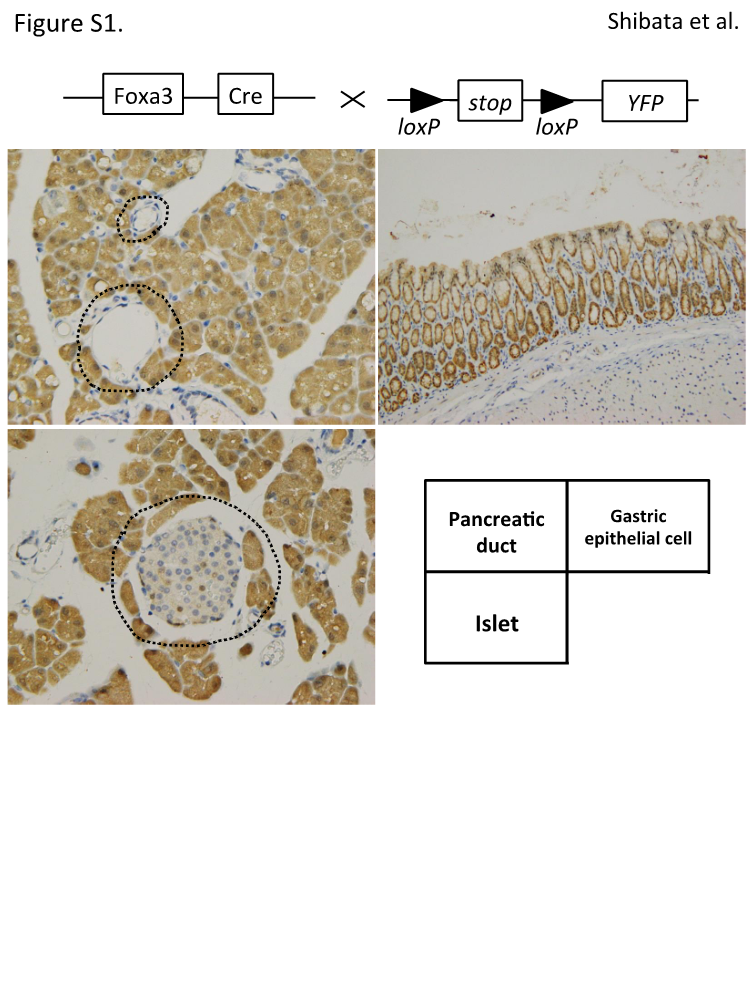


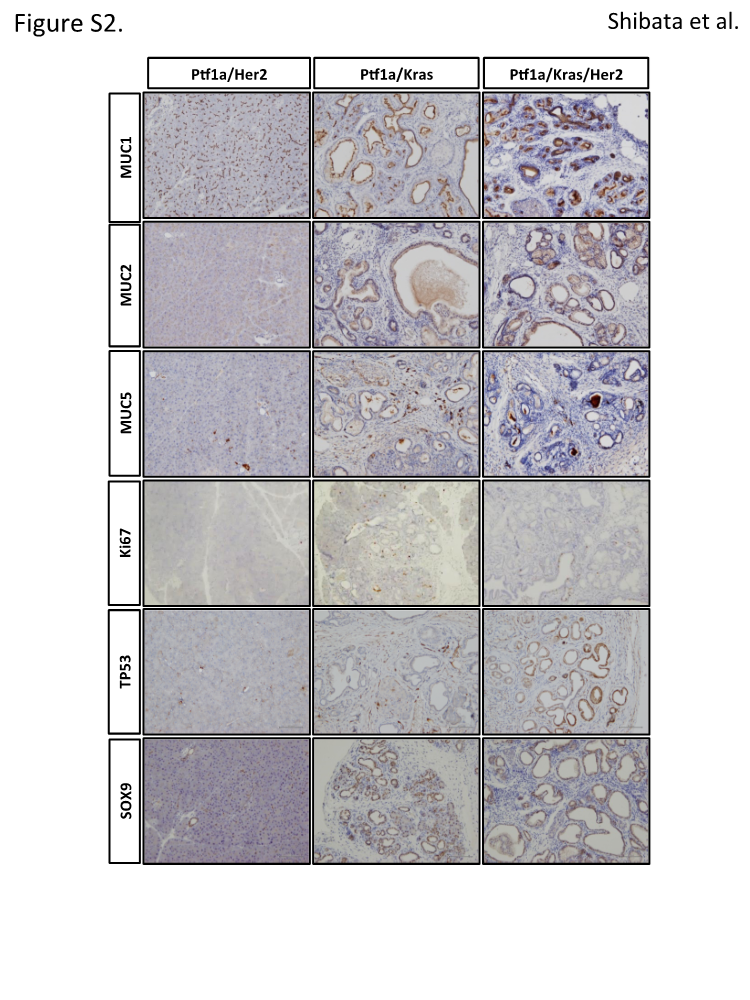


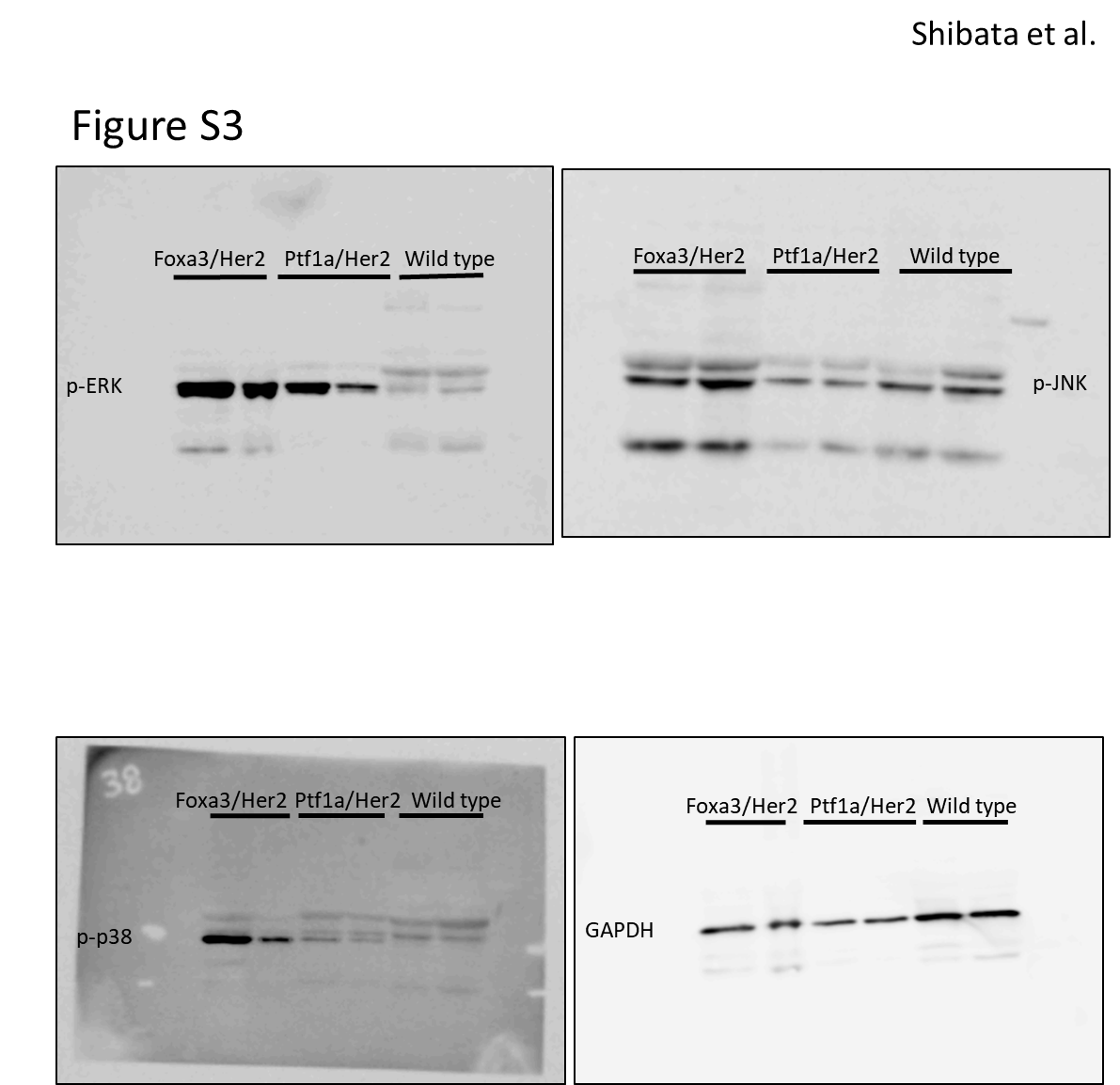


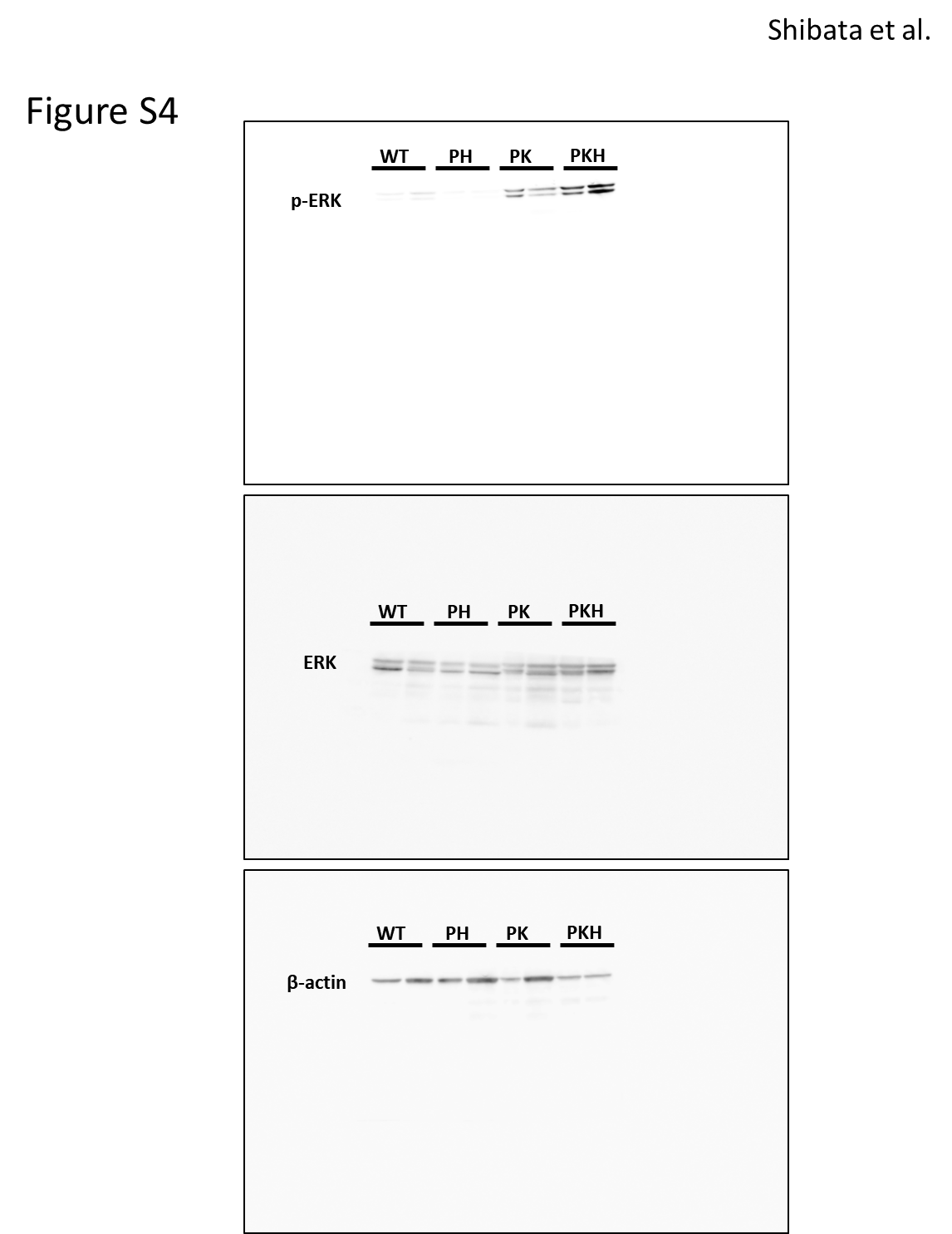


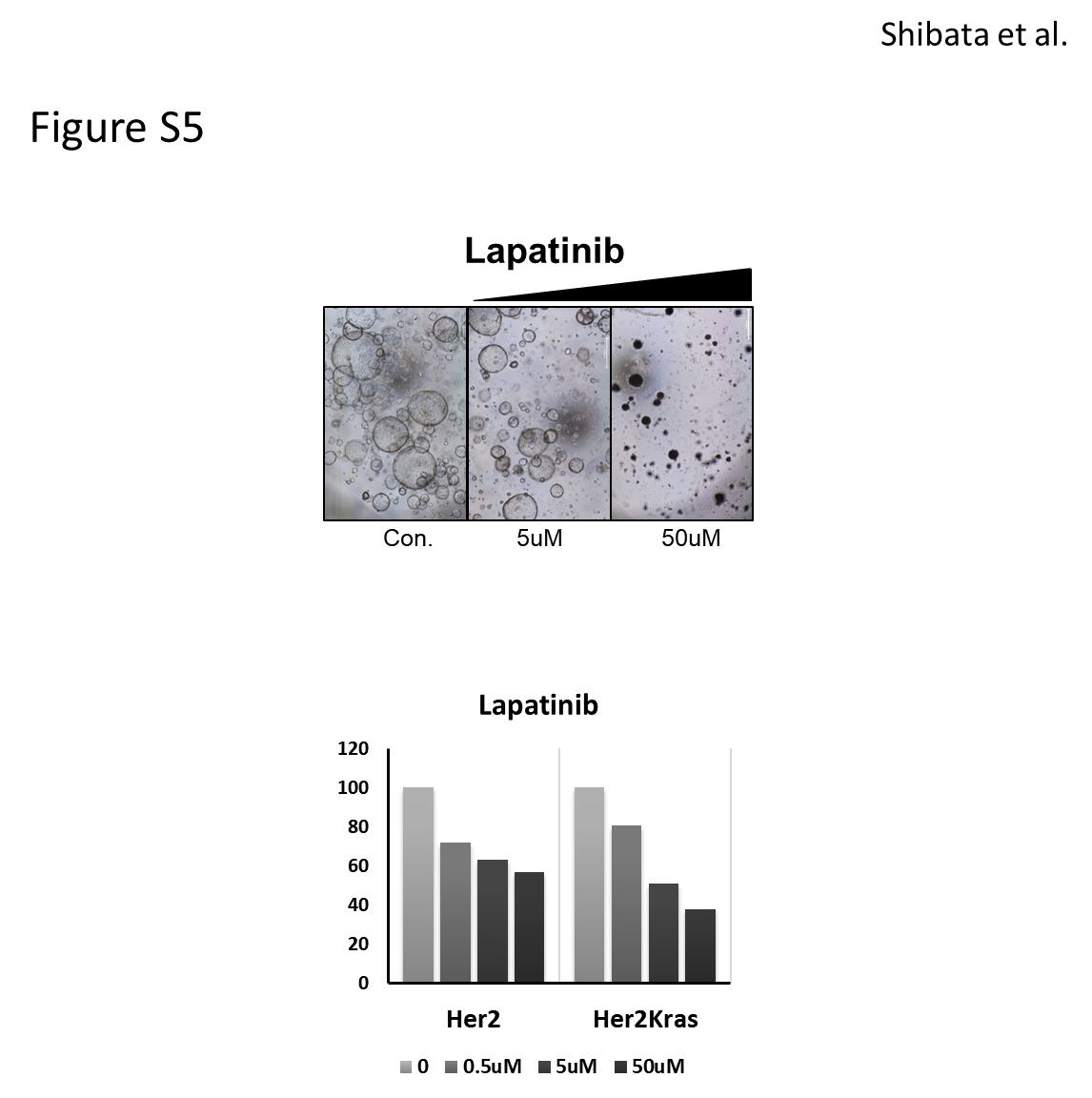


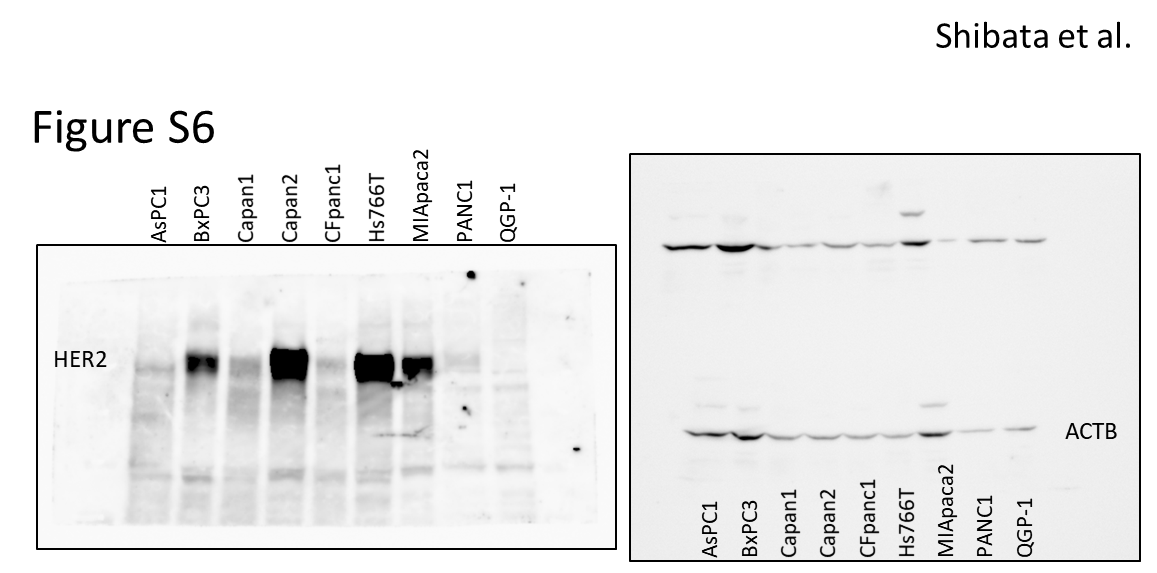


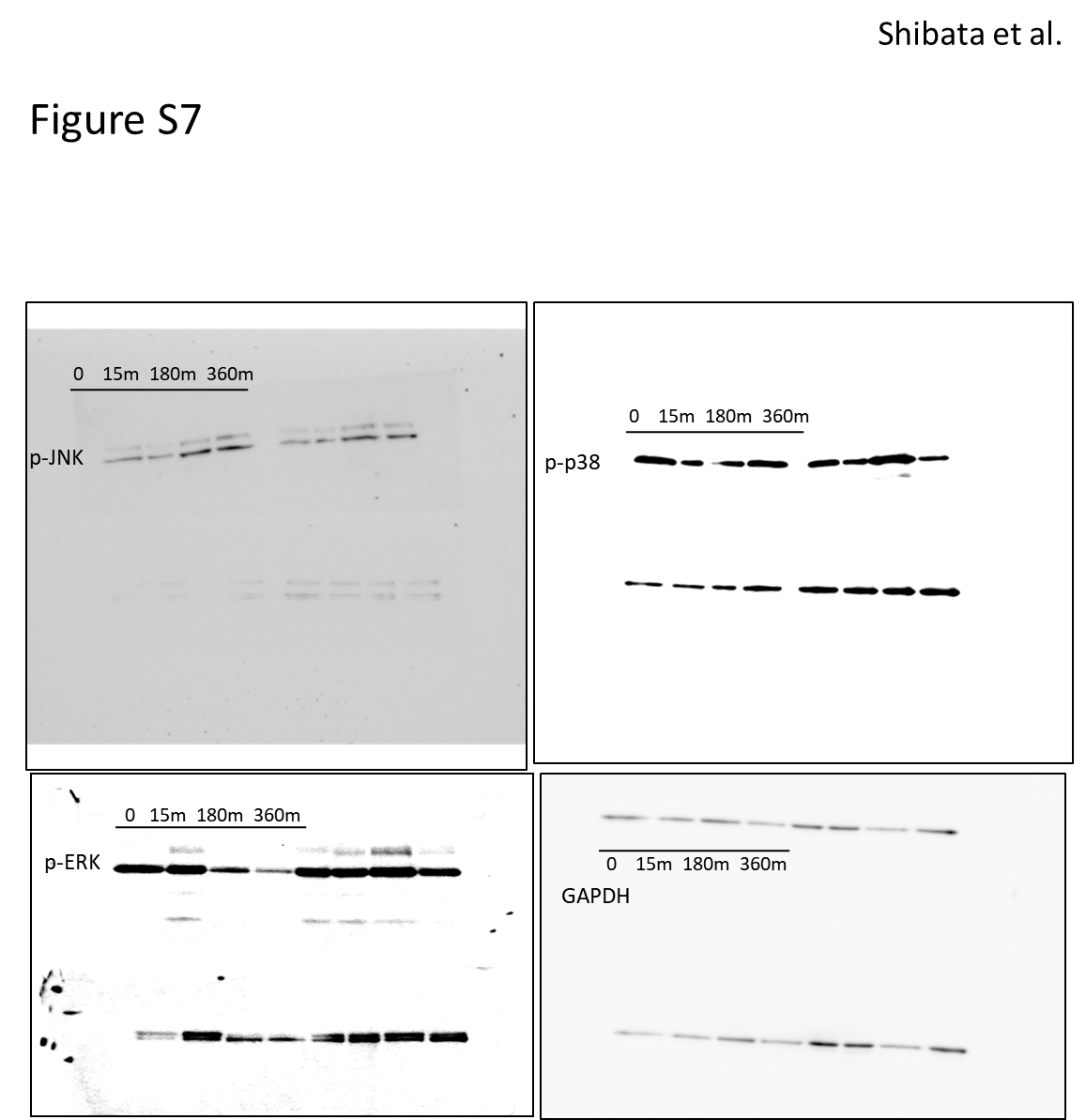

Supplement: Supplementary file 1 — Supplementary Information [file 41598_2018_24375_MOESM1_ESM.doc]
